# Vaginal microbiota and human papillomavirus: a systematic review

**DOI:** 10.4274/jtgga.galenos.2019.2019.0051

**Published:** 2020-09-03

**Authors:** Despoina Mortaki, Ioannis D. Gkegkes, Victoria Psomiadou, Nikos Blontzos, Anastasia Prodromidou, Fotis Lefkopoulos, Electra Nicolaidou

**Affiliations:** 1 First Department of Dermatology and Venereology, National and Kapodistrian University of Athens School of Medicine “A. Syggros” Hospital for Skin and Venereal Diseases, Athens, Greece; 2 Department of Surgery, Royal Devon and Exeter NHS Foundation Trust, Exeter, UK; 3 Clinic of Gynaecological Oncology, Metaxa Cancer Hospital, Piraeus, Greece; 4 Hellenic Society for Lower Genital Tract Disorders, Athens, Greece

**Keywords:** Microbiome, microbiota, human papillomavirus, HPV, Sneathia; Lactoba cillus gasseri, Lactobacillus jensenii, Lactobacillus crispatus

## Abstract

Accumulating evidence indicates the potential correlation between the vaginal microbioma and the acquisition and persistence of human papillomavirus (HPV) infection. This study aims to demonstrate the potential relationship through a systematic review of the current literature. A search was conducted on the following medical databases: PubMed and Scopus. Nineteen studies met the inclusion criteria and were incorporated in the present review. A total of 12.204 patients and their demographic characteristics were studied. Commercially available DNA tests and polymerase chain reaction (PCR) were used for the detection of different HPV subtypes, while the identification of the microbiomes was performed through specific diagnostic methods and PCR assay. The most frequently encountered species were classified based on their protective or detrimental impact on the progression of HPV infection. The beneficial role of some types of *Lactobacillus (Lactobacillus gasseri, Lactobacillus jensenii, Lactobacillus crispatus)* is generally supported. On the other hand, high microbial diversity and specific microorganisms such as *Sneathia, Anaerococcus tetradius, Peptostreptococcus, Fusobacterium* and *Gardnerella vaginalis* were found to be implicated with higher frequency and severity of disease, potentially resulting in pre-cancerous and cancerous cervical lesions.The role of vaginal microbiota appears to play an as yet not fully understood role in the susceptibility to HPV infection and its natural history.

## Introduction

Sexually transmitted diseases (STDs) are among the most frequent infectious diseases worldwide and are defined as those which include transmission of infectious organisms between sex partners. According to the Centers for Disease Control and Prevention almost 19 million cases are reported as infected each year by more than 20 different STDs (1).

Human papillomavirus (HPV) represents one of the most frequent causes of STDs in women around the world (2). More than 200 different HPV genotypes have been reported and are generally classified into two groups including high and low risk, which is based on the potential risk of developing cancer. To that end, in about 99% of all cervical malignancies one or more of the HPV types classified as high risk (16, 18, 31, 35, 39, 45, 51, 52, 56, 58, 59) are identified. Additionally, high risk types have been reported to play an important role in other malignancies such as anal, oropharyngeal, vulvar, and penile cancers. The genotype distribution, as well as the genome of each HPV, are considered critical for disease prevention, prognosis, and treatment (3,4).

The viral types of HPV can be easily transmitted from one person to another via skin and mucous membranes, which makes the possibility of infection from HPV relatively common. Nevertheless, the majority of infections are subclinical and transient, as they are suppressed by immunocompetent immune systems (5). Therefore, even though the incidence of HPV infection is common throughout life (>80% in sexually active people), the incidence of HPV- related diseases is lower (6).

Cervical cytology and HPV tests are widely used for cervical cancer screening and thus early detection of the underlying disease. Co-infections by multiple HPV types are likely to occur in more than 30% of HPV patients (2). Information regarding HPV co-infections can be an essential part of determining treatment options and therapy. Molecular diagnostic systems are capable of detecting more than 40 distinct HPV types, particularly those correlated to high grade dysplasia (HSIL). The human microbiome refers to the sum of microorganisms that may reside in various parts of the human body (eukaryotic, archae, bacteria and viruses), their genetic information and how they interact with the host environment (7). We now have a lot of data regarding the mapping of microbiota in several sites of human body, especially the gut, due to the development in sequencing technology. During the last years, there is emerging evidence that the vaginal microbiota may play a crucial role in HPV carcinogenesis (8) and is related to protection against dysbiosis and HPV infection (9,10). In healthy women of reproductive age, vaginal pH is primarily regulated by lactic acid producing bacteria such as *Lactobacillus* species. In women, whose vaginal microbiota is not lactobacilli-dominated, anti-bacterial defensive mechanisms are decreased (11). Alterations in vaginal microbiota, such as bacterial vaginosis and vaginal infections, are usually correlated to respective changes in vaginal pH. In this setting a decrease in vaginal pH has been related with decreased risk of infections, such as *Chlamydia trachomatis*, trichomoniasis, and urinary tract infections. In the vaginal environment, five major community state types (CST) were described by Ravel et al. (12) (CST I-V), who studied the vaginal microbiota of 396 asymptomatic women and characterized the found species in five groups based on their genes. In a healthy environment, the following microorganisms are recognized; CST I, II, III, V and are dominated by *Lactobacillus crispatus (L. crispatus), Lactobacillus gasseri (L. gasseri), L. iners and Lactobacillus jensenii (L. jensenii)*. respectively. In contrast, CST IV is characterized by depletion of lactobacilli and increased diversity of anaerobic bacteria, such as *Atopobium* (12). Nevertheless, there are many questions yet to be answered concerning the relationship between the vaginal microbiota and how it correlates with the HPV natural history.

The aim of the present study was to present the current knowledge and to evaluate the correlation between microbiome and HPV.

## Data sources

An extensive systematic search was performed in both PubMed and Scopus. All databases were searched up to February 25, 2019. The search strategy used in both databases included the combination of the key words: (microbiome or microbiota) and (HPV or human papillomavirus). The references of relevant articles were also hand-searched, for additional studies.

## Study selection criteria

Studies reporting data on the association of microbiota and HPV were included in this systematic review. Abstracts in scientific conferences, editorials, and reviews as well as animal studies were not included in the study. Studies published in languages other than English, Dutch, German, Greek, Italian or Spanish were not taken into consideration.

## Selected studies

**Figure 1:**
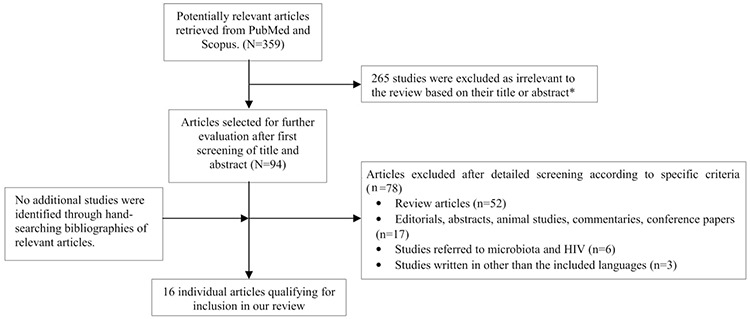
Flow diagram of the selection process of articles included in the review HIV: Human immunodeficiency virus *The majority of the studies were retrieved in both databases

A total of 78 and 291 articles were retrieved from PubMed and Scopus respectively. From those studies, 16 studies were identified as eligible for inclusion in this review. No additional studies were identified through hand-searching of references. The included studies are graphically presented in Figure 1 (flow diagram).

## Techniques

From the eligible articles, the techniques that were used for HPV detection and the identification of species present in the microbiome were DNA tests, Sequencing and polymerase chain reaction (PCR) amplification.

## Human papillomavirus detection

HPV detection and genotyping was performed either with commercially available DNA tests, such as Roche Linear Array HPV Genotyping test and Digene Hybrid Capture II DNA test, or through an assay of PCR using specific primers such as MY09/MY11 or GP5+/GP6+. A range of 15-49 HPV types was identified, including predominantly high-risk HPV types with or without low risk subtypes (13,14).

## Vaginal microbiota

For the detection of vaginal microbiota, the initial assessment included diagnostic methods such as microscopic evaluation, Gram stain test, microbiological cultures and measurement of vaginal pH. Amsel criteria were used for diagnosing bacterial vaginosis (15). PCR amplification of V1-V5 hypervariable region of the 16S rRNA genes was used in 12 studies. PCR results were analyzed in accordance with databases such as BLAST, QIIME and Illumina MiSeq in order to identify specific species (Table 1).

## Study characteristics

Sixteen studies, including a total of 12.204 patients, have been published in the literature with regard to the association between vaginal microbiota and HPV infection (16,17,18,19,20,21,22,23,24,25,26,27,28,29,30,31,32,33,34).

According to the searched literature, participants were categorized based on several epidemiologic criteria which included age, race/ethnicity, educational status, marital status, time of first intercourse, lifetime number of sexual partners, number of live births, use of hormonal contraception or antibiotics/probiotics, prevalence of smoking, alcohol use, and menstrual phase.

Concerning specific population characteristics, the correlation between HPV infection and vaginal microbiota has been studied in human immunodeficiency virus (HIV) infected patients (24,28), while Lee et al. (29) studied a twin cohort, as well as their siblings and mothers (18). A total of 824 individuals were reported to be female sex workers (17,29). Moreover, Kero et al. (31) enrolled 329 asymptomatic, pregnant women in the third trimester of their pregnancy and they studied the potential impact of vaginal microbiota on the outcome of known HPV infection during a 72-month follow-up. Brotman et al. (22) conducted a study of 32 patients, in which they assessed 937 self-collected samples before and after vaginal douching cessation.

The demographic details of the populations under study are shown in Table 2.

## Types of vaginal microbiota

The detected microbiotas in these studies included several types of microorganisms: *L. iners* classified as CSTIII (13 studies, 72.22%); *L. crispatus* classified as CSTI (8 studies, 44.44%); CSTIV-B which represents anaerobic microbiomes combined with reduced *Lactobacillus* (5 studies, 27.77%); *Megasphaera, G. vaginalis* and *L. jensenii* which is classified as CSTV (4 studies, 22.22%); *Sneathia, L. gasseri* classified as CSTII as well as CSTIV-A which represents *Peptoniphilus, Anaerococcus, Corynebacterium, Finegoldia* and *Prevotella* (3 studies, 16.66 %); *Prevotella, L. vaginalis, Phylum protebacteria, Lactobacillus reuteri (L. reuteri)* and other members of Pseudomonas family (2 studies, 12.5%); and in one study each (6.25%) *Dialister, L. formicalis, Fusobacterium, Lactobacillus gallinarum* and *Lactobacillus salivarus* (found only among South African women).

## Vaginal microbiota association with HPV and CIN

Interestingly, it was found that women with HPV infection had a higher diversity and a lower proportion of *Lactobacillus*. A lower prevalence of *L. iners* and *L. crispatus* was also observed (20). Dols et al. (16) also reported a shift of the composition of the vaginal lactobacilli in HPV (+) women as well as a significantly reduced prevalence of *L. crispatus*. Other common microorganisms among HPV (+) patients were *L. gasseri* and *Gardnerella vaginalis* (17).

Additionally, patients who were eventually diagnosed with a cervical intraepithelial neoplasia (CIN) also had a high diversity of their vaginal microbiota (25) and they were usually colonised by *Sneathia*, while in women with invasive cervical cancer, *Fusobacterium* was the most common type of microorganism (26). Interestingly, Piyathilake et al. (27) found an abundance of *Lactobacillus* and *L. reuteri* specifically in women with CIN II.

In contrast, in HPV (-) women, *L. crispatus*/CSTI and *L. gasseri*/CSTII were the most common species (26). *L. crispatus* was related by Reimers et al. (28) and Borgdorff et al. (21) to decreased prevalence of oncogenic HPV types, while Darend identified prevalent high risk HPV infections among women with a decreased population of *Lactobacillus* and an increased abundance of anaerobes, particularly of the genera *Prevotella* and *Leptotrichia* (19,22).

## Vaginal microbiota and HPV remission

The relationship between vaginal microbiota and HPV remission was highlighted by Brotman et al. (22). CSTIII was the classification group with the fastest remission, while CST IV-B was the one with the slowest. This was also confirmed by Di Paola et al. (32), who suggested CSTIV-B to be a risk factor for HPV persistence.

## Transition to HPV and severity of infection

Brotman et al. (22) compared the CSTs among women HPV (-) who later became HPV (+) and reported that CSTIV-A was related to higher transition to HPV (+) status than CSTI.

Concerning the severity of the HPV infection, Mitra et al. (25) reported CSTIV and CSTV to be associated with high severity when CSTI was associated with low severity. CSTIII was related by Piyathilake et al. (27) to high severity CIN lesions.

## Vaginal microbiota, HIV and HPV infections

Five of the above mentioned studies have shown an association between vaginal microbiota, HPV and HIV infection. *L. crispatus* was found to have an advantageous effect on the HPV infection evolution in both HIV-infected and uninfected women and in general *L. crispatus* was found to be a protective factor against HIV, high risk HPV and Herpes Simplex type 2, as it was found in high abundance in uninfected women while, in contrast, infected women had a reduced prevalence. In both HIV and HPV infections, a comparable shift in the composition of the *Lactobacillus* flora was identified.

## Vaginal microbiota, ethnicity and HPV infection

Another factor that strongly affects the vaginal microbiota seems to be ethnicity. The studies included in this review refer to women of all ethnicities: European/Caucasian, Asian, Latin-American and African. It has been found that Afro-Caribbean women have a fourfold higher risk of suffering from a vaginal dysbiosis or high microbiota diversity, which indicates that the most common type of microbiota among them is CST IV, in comparison with European/Caucasian and African women. However, the prevalence of HPV and the rate of more severe lesions was not proportionately higher (35).

## Microbiological markers of HPV infection

It is remarkable, that among all the microbiota, Fusobacteria including *Sneathia*, were identified as a possible microbiological marker correlated with HPV infection, as was shown by Lee et al. (29). However, the relation between HPV infection and the coexistence with other types of vaginal microbiota appears to be either protective, or to predispose to HPV infection. In addition, the evolution of HPV infection is in direct correlation with the species or genus of the vaginal microbiota dominating the vaginal environment. Specifically, some types of *Lactobacillus* including *L. gasseri, L. jensenii* and *L. crispatus* seem to protect from HPV infection while on the contrary other microorganisms, especially *Sneathia, Anaerococcus tetradius, Peptostreptococcus, Fusobacterium, Gardnerella vaginalis* and *L. iners*, often together with a low abundance of the other types of *Lactobacillus* and other factors such as smoking and lack of barrier contraception or low estrogen levels, not only lead to elevated rates of HPV infection, but also to higher disease severity and lower HPV remission. This suggests that some of the microbiota species may be used as a disease marker or even as a therapeutic mean against HPV. The effect of the vaginal microbiota on the evolution of an HPV infection is described in Table 3.

## Discussion

A systematic review of the literature was conducted with the aim of examining if vaginal microbiota composition patterns can be related to HPV infection and intraepithelial lesions. This study indicates a significant correlation among vaginal microbiota and HPV infection in that certain microbial species appear to play a protective role against HPV infection while others predispose to either the progression or the remission of the disease.

Alterations in vaginal microbiota have been associated with a variety of complications, either obstetric or gynecologic, such as tubal factor infertility, spontaneous abortion, intrauterine fetal demise, premature rupture of membranes, pre-term labor and delivery, intrauterine growth restriction, endometritis, postpartum infection, chorioamnionitis, ectopic pregnancy and pelvic inflammatory disease (35). Some of these conditions may cause chronic and severe congenital tract infections, which have been associated with vaginal flora alterations (36). Despite the advances in molecular elucidation of the vaginal microbiota, the exact pathophysiologic pathway has not yet clearly identified.

Microbiota analysis of HPV positive patients and patients with CIN revealed significant alterations compared to that present in women without either HPV positivity or CIN. Certain microbiotic species found in the vagina have been associated with increased risk of infection from HPV and can serve as a critical predictive and prognostic markers for the early detection of those pathologies. For example, a significant proportion of *Lactobacillus* species have been documented to be protective against HPV whereas *Sneathia* species can negatively affect the evolution of HPV. This is in accordance with respective studies in the field which showed that decrease in *Lactobacillus* species in vaginal flora, had significant impact on eubiosis and led to increase in concentration of pathogenic anaerobic bacteria such as *Gardnerella* and *Sneathia* (37). Furthermore, remission and severity of HPV infection were additionally influenced by the presence of certain microbiotic species in the vaginal environment. Moreover, there appears to be a strong association between dysbiosis protection and HPV infection, even though the pathophysiology of this association is not yet fully understood. It has been proposed that these mechanisms not only encompass the patient’s defensive function but also her past immunological response against HPV (38). The potential impact of dysbiosis on the immune system could explain the susceptibility of those women to HIV infections (39).

The association between ethnicity and HPV with regards to the vaginal microbiota has not yet been clearly determined. This review also revealed that Afro-Caribbean ethnicity was associated with alterations in vaginal flora. However, HPV incidence was not significantly different in Afro-Caribbean women with dysbiosis. On the contrary, according to a recent meta-analysis, African women presented with higher rates of HPV infection compared to other ethnicities (40). Nonetheless, research in these populations is still limited and further studies are needed so as to elucidate the association among dysbiosis and HPV infection in African populations. Another factor that should be addressed is that flora alterations are responsible not only for increased susceptibility to HPV infections, but are also present in cancerous and pre-cancerous cervical disorders (8). More specifically, vaginal dysbiosis has been associated with more rapid disease progression and more advanced disease stages (25).

Several limitations can be found in such a newly studied field. First and foremost, the number of studies and thus the number of patients included is limited, which serves to highlight the innovation of this approach. In terms of search strategy used for this review, it could be considered unduly limited due to the exclusion of abstracts, review articles, conference papers, editorials, animal studies, and commentaries. The retrospective nature of the available studies could also be highlighted. Large prospective randomized controlled trials are necessary to clarify the possible correlation of vaginal microbiota with HPV and related pathologies.

## Conclusion

Recently available data suggest a potential association between the vaginal microbioma and HPV infection. Specifically, (i) highly diverse vaginal flora, (ii) identification of specific species such as *Sneathia*, (iii) low concentration of *Lactobacillus* and the subsequent vaginal dysbiosis were found to affect the incidence, persistence and severity of HPV infection. The aforementioned parameters should be subjected to further investigation via multicentre trials in order to be validated as robust independent risk factors.

## Figures and Tables

**Table 1:**
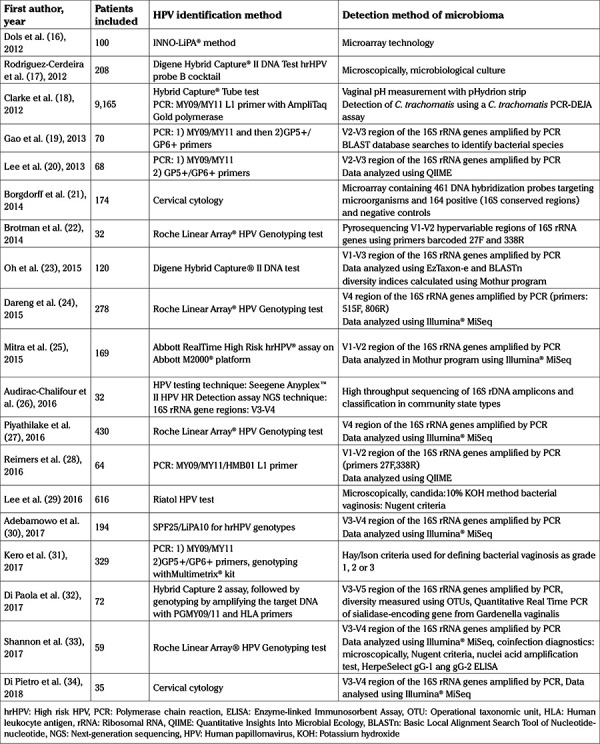
Summary of the techniques applied in the included studies

**Table 2:**
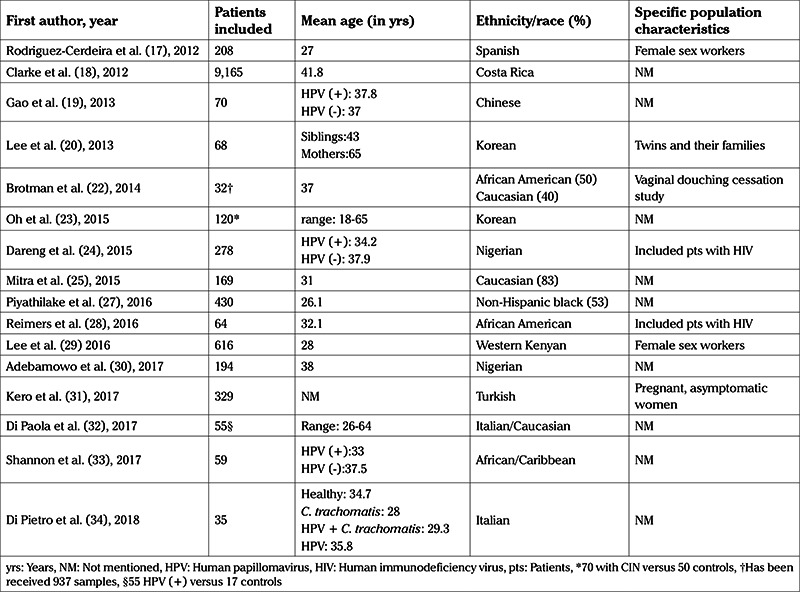
Demographics of the populations in the included studies

**Table 3:**
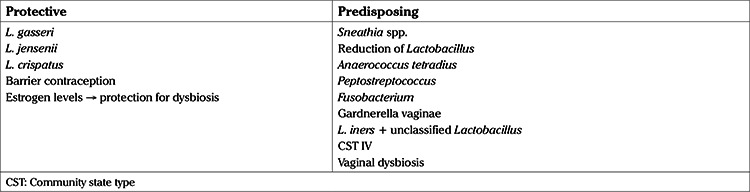
Protective and burdening parameters of vaginal microbiota regarding human papillomavirus infection/persistence.
